# Incidence trends and spatial distributions of lung adenocarcinoma and squamous cell carcinoma in Taiwan

**DOI:** 10.1038/s41598-023-28253-4

**Published:** 2023-01-30

**Authors:** Hsin-I Liu, Chun-Ju Chiang, Shih-Yung Su, Jing-Rong Jhuang, Dai-Rong Tsai, Ya-Wen Yang, Li-Ju Lin, Yu-Chen Wang, Wen-Chung Lee

**Affiliations:** 1 Institute of Epidemiology and Preventive Medicine, College of Public Health, National Taiwan University, Taipei, Taiwan; 2 Taiwan Cancer Registry, Taipei, Taiwan; 3 Master Program in Statistics, National Taiwan University, Taipei, Taiwan; 4 Health Promotion Administration, Ministry of Health and Welfare, Taipei, Taiwan; 5 Institute of Epidemiology and Preventive Medicine, College of Public Health, National Taiwan University, Rm. 536, No. 17, Xuzhou Rd., Taipei, 100 Taiwan

**Keywords:** Diseases, Oncology, Mathematics and computing

## Abstract

Lung cancer is the second most common cancer in Taiwan. After Taiwan implemented the Tobacco Hazards Prevention Act in 1997, smoking rates declined. However, the incidence rates of lung cancer for both sexes are still increasing, possibly due to risk factors other than smoking. We used age–period–cohort analysis to examine the secular trends of lung cancer incidence rates by histological type in Taiwan. A stabilized kriging method was employed to map these lung cancer incidence rates. Lung adenocarcinoma incidence rates increased, but lung squamous cell carcinoma incidence rates decreased, for both the sexes in recent birth cohorts, particularly in women. In Taiwan, the hotspots of lung adenocarcinoma incidence rates were in the northern, northeastern, and western coastal areas; the incidence rates increased rapidly in the western and southern coastal regions and southern mountainous regions. The high incidence rates of lung squamous cell carcinoma in men were in the southwestern and northeastern coastal areas. The incidence rates rapidly increased in the central and southern coastal and mountainous regions. For both sexes in Taiwan, lung squamous cell carcinoma incidence rates declined from 1997 to 2017, but lung adenocarcinoma increased. The increased incidence rates of lung adenocarcinoma may be related to indoor and outdoor air pollution. Some areas in Taiwan have increasing lung cancer incidence rates, including the northwestern and southern coasts and mountains, and warrant particular attention.

## Introduction

Lung cancer is the second most common cancer and a leading cause of cancer deaths worldwide. In 2020, an estimated 2.2 million new lung cancer cases developed globally, accounting for 11.4% of all cancer cases^1^. Lung cancer incidence varies worldwide. In most developed countries in Europe and North America, the lung cancer incidence rate in men has decreased, albeit slowly, but has increased in women. Tobacco has been classified as a carcinogen by the International Agency for Research on Cancer^2^. Smoking, a major risk factor for lung cancer, mainly causes squamous cell and small cell carcinomas and slightly increases adenocarcinoma risk^3–5^.

In Taiwan, lung cancer is the third most common cancer. The age-standardized incidence rate was 43.5 per 100,000 in men and 31.6 per 100,000 in women in 2017^6^. In 1997, Taiwan implemented the Tobacco Hazards Prevention Act. From 1997 to 2017, the smoking rate decreased from 55.1 to 26.4% in adult men and from 3.3 to 2.3% in adult women^7^. More than half of patients with lung cancer are nonsmokers^8^. Therefore, risk factors including environmental tobacco smoke (ETS), environmental pollution (e.g., air pollution, radon, and arsenic), and occupational pollution (e.g., asbestos) warrant investigation^9,10^.

This study examined the incidence trends and spatial distributions of lung cancer and its histological types in Taiwan using age–period–cohort analysis. In addition, we used a stabilized kriging method to map lung cancer incidence rates in Taiwan.

## Methods

Data on incident lung cancer cases were obtained from the Taiwan Cancer Registry^11,12^, a nationwide population-based registry that provides critical data on cancer incidence, care, and survival in Taiwan. The registry has collected the data of patients with newly diagnosed cancer from hospitals with ≥ 50 beds in Taiwan since 1979. Each year, qualified cancer registrars at the reporting hospitals identify new cancer cases and submit the data (in the form of individual records) to the central office of the registry. Since 1997, the completeness, quality, and timeliness of Taiwan Cancer Registry data have been greatly improved. Some quality indicators for this registry are as follows: population coverage = 98.4%; percentage of cases with death certificate only = 0.9%; percentage of morphological verification = 93.0% for all sites combined; data timeliness = 14 months, which ranks the registry among the top-tier gold-level cancer registries in the world.

In the registry, lung cancer is identified by its *International Classification of Diseases for Oncology, Field Trial Edition* (*ICD-O-FT*) topography code (i.e., 162) for cases before 2002 and its *International Classification of Diseases for Oncology, Third Edition* (*ICD-O-3*) code (i.e., C33-C34) for cases after that. According to morphology codes, lung cancer was classified into five histological types (Supplement 1): adenocarcinoma, squamous cell carcinoma, large cell carcinoma, small cell carcinoma, and other specified or unspecified carcinoma. Population numbers were obtained from the online database provided by the Department of Statistics of Taiwan’s Ministry of the Interior. The variables analyzed in this study included sex, age, and administrative area.

We calculated age-standardized lung cancer incidence rates using the World Health Organization (WHO) 2000 Standard. We used average annual percent change (AAPC)^13^ to investigate the incidence trends from 1997 to 2017. We performed age–period–cohort analysis of the incidence rates from 1997 to 2016 for only adenocarcinoma and squamous cell carcinoma because the numbers of other subtypes were too low. We categorized the data into eleven 5-year age groups (30–34, 35–39, …, 80–84 years) and four 5-year periods (1997–2001, 2002–2006, 2007–2011, and 2012–2016), resulting in a total of 44 cross-classified age–period cells. Patients aged < 25 or > 85 years were not included because relevant cases were scarce and defining a midpoint was difficult. By subtracting the median age at diagnosis from the middle of each calendar period for each cell, we obtained 14 cohorts separated by 5-year intervals based on the median year of birth (1917, 1922, …, 1982). The age–period–cohort method is unidentified because the relationships among the three temporal variables are perfectly linear (i.e., cohort = period–age). We imposed the constant relative variation assumption^14^ to circumvent this problem, specifying that age effects are deterministic whereas period and cohort effects are stochastic, with a constant relative variation.

We mapped lung cancer risk for 349 administrative areas on the main island of Taiwan (excluding the 19 administrative areas on the offshore islands). Mapping was performed only for adenocarcinoma and squamous cell carcinoma because of the scarcity of other lung cancer types. We used the standardized incidence ratio (SIR) to represent lung cancer risk in an administrative area. The standard rates used for calculating SIRs were the average age-specific incidence rates from 2013 to 2017 stratified by histological type and sex. Administrative areas with SIRs of > 1 (i.e., risk greater than that of the whole island), nearly 1 (i.e., risk similar to that of the entire island), and < 1 (i.e., risk lower than that of the whole island) were respectively indicated by red, white, and blue shading on the map. AAPC was also used to measure the risk trend in each administrative area from 1997 to 2017 (Supplement 2). Administrative areas with risk trending upward (AAPC > 0), remaining stable (AAPC ≈ 0), and trending downward (AAPC < 0) were respectively indicated by red, white, and blue shading on the map. Finally, we used a kriging method^15,16^ to stabilize the SIR and AAPC values and plotted the smooth curve with contour lines.

### Ethics approval and consent to participate

This study protocol was approved by the National Taiwan University Research Ethics Committee (202101HM030) and the Data Release Review Board of the Health Promotion Administration, Ministry of Health and Welfare in Taiwan. All methods were performed in accordance with the relevant guidelines and regulations. In addition, the National Taiwan University Research Ethics Committee waived the requirement for informed consent due to the lack of personal information and secondary data in the study.

## Results

**Figure 1:**
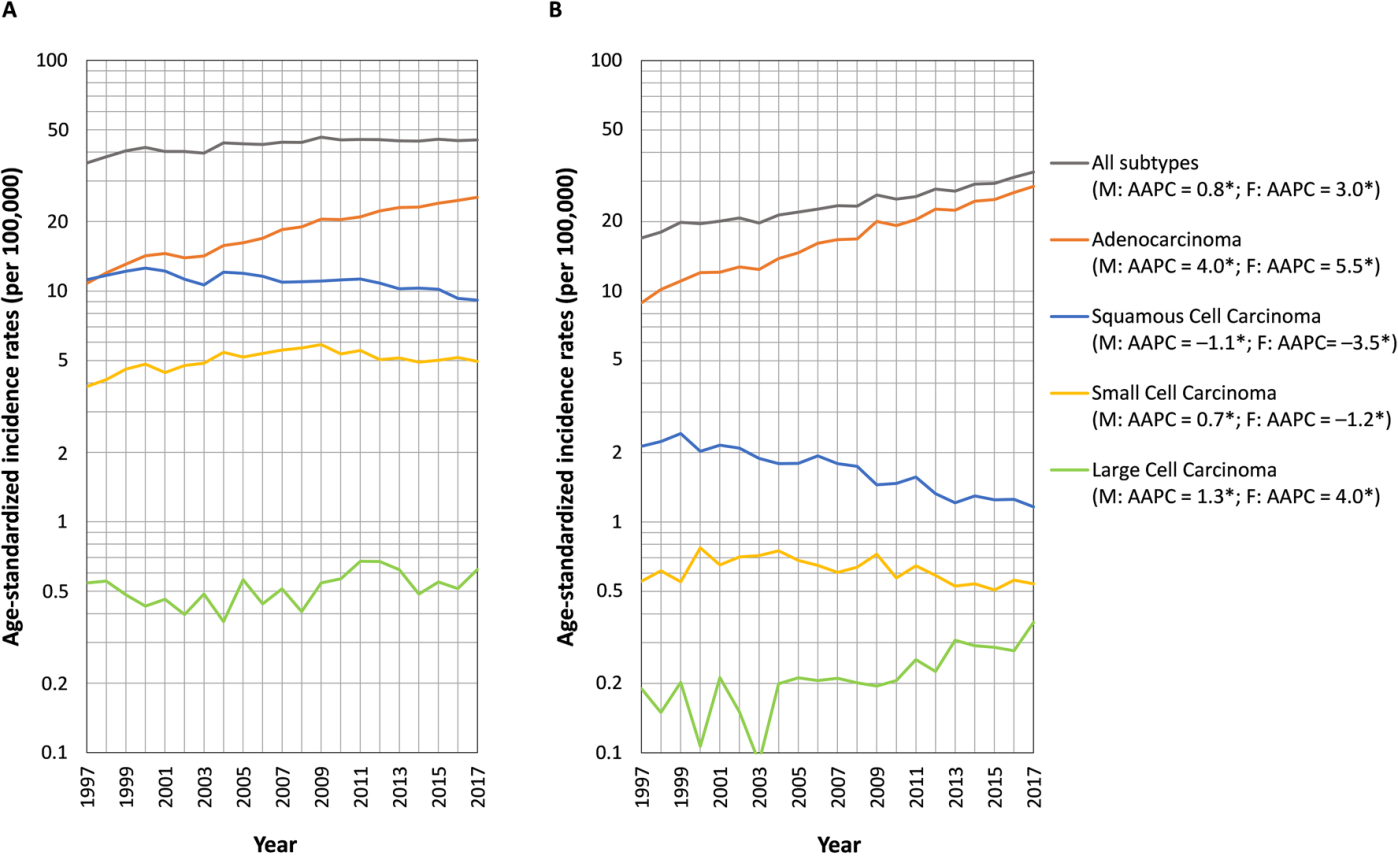
Age-standardized incidence rates of lung cancer and its subtypes from 1997 to 2017 (**A)** males; (**B)** females. *: average annual percent change (AAPC) is significantly different from zero.

Figure 1 shows the trends in the age-standardized incidence rates of lung cancer from 1997 to 2017 in Taiwan. In men, the overall lung cancer incidence rate increased, but this increase slowed near the end of the study period (AAPC = 0.8, 95% confidence interval [CI]: 0.6, 1.1). In women, the rate rapidly increased without interruption (AAPC = 3.0, 95% CI 2.8, 3.3). Adenocarcinoma incidence increased the fastest among the histologic types, particularly in women; the rate in men increased from 10.8 to 25.5 per 100,000 individuals from 1997 to 2017 (AAPC = 4.0, 95% CI 3.7, 4.3), and women, it increased from 8.9 to 28.5 per 100,000 individuals from 1997 to 2017 (AAPC = 5.5, 95% CI 5.2, 5.8). The incidence rates of squamous cell carcinoma decreased, particularly in women; the rate in men decreased from 11.2 to 9.1 per 100,000 individuals from 1997 to 2017 (AAPC =  − 1.1, 95% CI − 1.5, − 0.8), and in women, it decreased from 2.1 to 1.2 per 100,000 individuals (AAPC =  − 3.5, 95% CI − 4.0, − 3.0). The incidence rate of small cell carcinoma in men increased first and then decreased slightly, but the overall trend was an increase (AAPC = 0.7, 95% CI 0.0, 1.4); in women, the rate was low, at approximately 0.6 per 100,000 individuals, and decreased (AAPC =  − 1.2, 95% CI − 2.0, − 0.4). The incidence rate of large cell carcinoma in men was also low, at approximately 0.5 per 100,000 individuals, but it increased (AAPC = 1.3, 95% CI 0.2, 2.4); in women, the rate was even lower at approximately 0.2 per 100,000 individuals, but it substantially increased in recent years (AAPC = 4.0, 95% CI 2.6, 5.4). Supplement 3 presents the proportions of lung cancer histologic types from 1997 to 2016: Adenocarcinoma was the most common histologic type, with its proportion increasing to nearly 90% in women and > 50% in men. Squamous cell carcinoma was another major type in men but was less common in women, and its proportions declined annually for both sexes. The proportions of small cell carcinoma, large cell carcinoma, and other specified or unspecified carcinoma in men and women were meager and declined.

**Figure 2:**
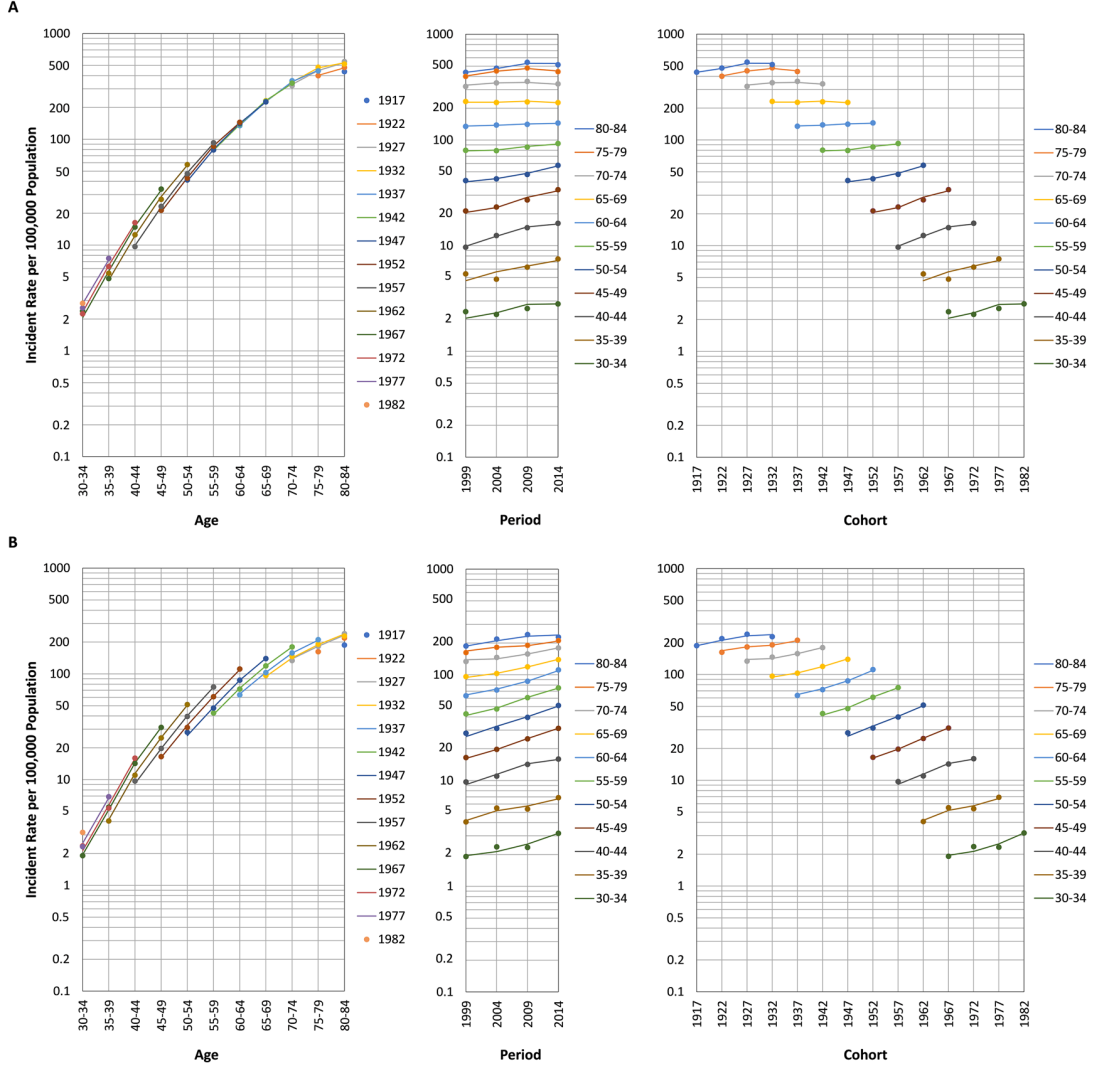
Incidence rates of lung cancer by age, period, and cohort (**A)** males; (**B)** females.

Figure 2 presents the lung cancer incidence rates by age, period, and cohort. The incidence rates increased with age, but this increase slowed with age. In men, the temporal incidence rate trends for age groups were diverse; the rates increased but became flat or declined after 2009 for age groups of 70–74, 75–79, and 80–84, were flat for age groups of 60–64 and 65–69, increased monotonically in younger ones (35–39, 40–44, 45–49, 50–54, and 55–59 years), and increased but slowed down after 2009 in the youngest (30–34 years). In women, the incidence rate in the oldest age group (80–84 years) increased in earlier periods before flattening out in recent periods. In contrast, the rates in the other age groups continued to grow. The incidence rate trends for birth cohorts were more consistent in different ages for both sexes. In men, the rates for earlier birth cohorts increased. The rates in 1932 to 1957 birth cohorts remained stable, whereas those in 1957 to 1967 increased rapidly. The rate increase slowed down in the 1967 to 1977 birth cohorts; in later cohorts, the rates plateaued. In women, the rates in the earlier birth cohorts also increased monotonically. The rates for the 1932 to 1967 birth cohorts increased rapidly, those for the 1967 to 1977 birth cohorts increased slowly, and those for the post-1977 cohorts quickly increased. Supplements 4 and 5 present the incidence rates by age, period, and cohort for lung adenocarcinoma and lung squamous cell carcinoma, respectively. Supplement 6 presents the AAPC in the incidence rates in various age groups for lung cancer, lung adenocarcinoma, and lung squamous cell carcinoma, respectively.

**Figure 3:**
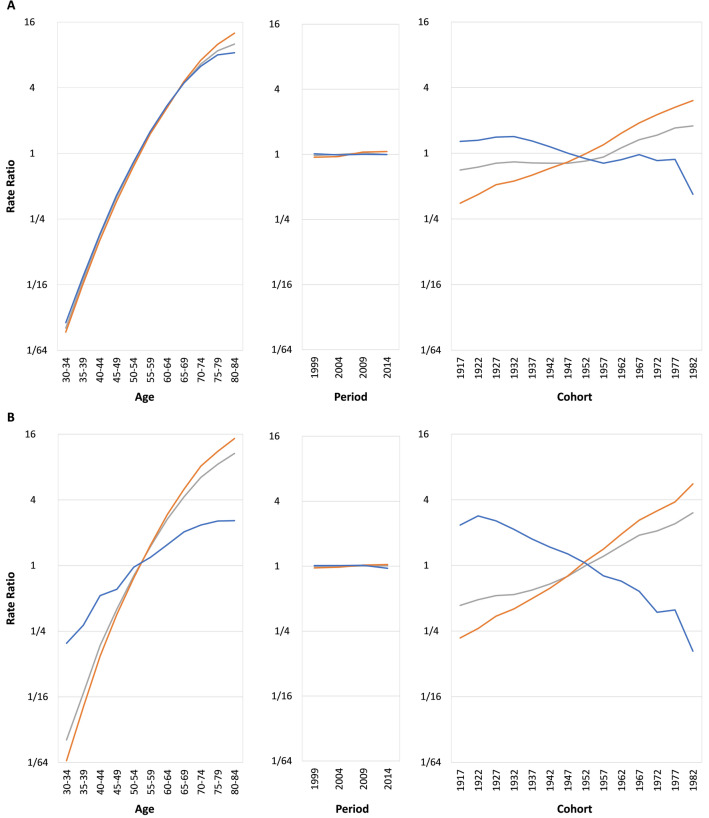
Age, period, and cohort effects of lung cancer, lung adenocarcinoma, and lung squamous carcinoma (**A)** males; (**B)** females; GRAY: lung cancer; ORANGE: lung adenocarcinoma; BLUE: lung squamous cell carcinoma).

Figure 3 shows our analysis’s age, period, and cohort effects. Lung cancer risks increase with age for both sexes, but these increases were slower in older age groups (and in middle age groups for lung squamous cell carcinoma in women). The period effects of lung cancer were flat for both sexes. For the cohort effects of lung cancer, the rates increased in earlier cohorts for both sexes, whereas they decreased slightly before rising in men but increased consistently in women in the 1932 to 1967 birth cohorts. The rates increased slowly for both sexes in the 1967 to 1977 birth cohorts; finally, the rates plateaued in men but increased in women in later cohorts. The age effects of lung adenocarcinoma for both sexes were similar but more prominent than overall lung cancer. Lung adenocarcinoma increased slightly for both sexes over time. The cohort effects of lung adenocarcinoma increased for both sexes; they increased rapidly for cohorts born after 1977 in women. The age effect of lung squamous cell carcinoma increased with age in men but increased slowly in older age groups, and that in women also increased but less so, and the increase started to slow in the middle age groups. The period effects of lung squamous cell carcinoma were almost flat for both sexes but decreased slightly in later periods in women. Regarding the cohort effects of lung squamous cell carcinoma, the rates increased in early birth cohorts for both sexes but decreased in men starting with the 1932 birth cohort and in women starting from the 1922 birth cohort.

**Figure 4:**
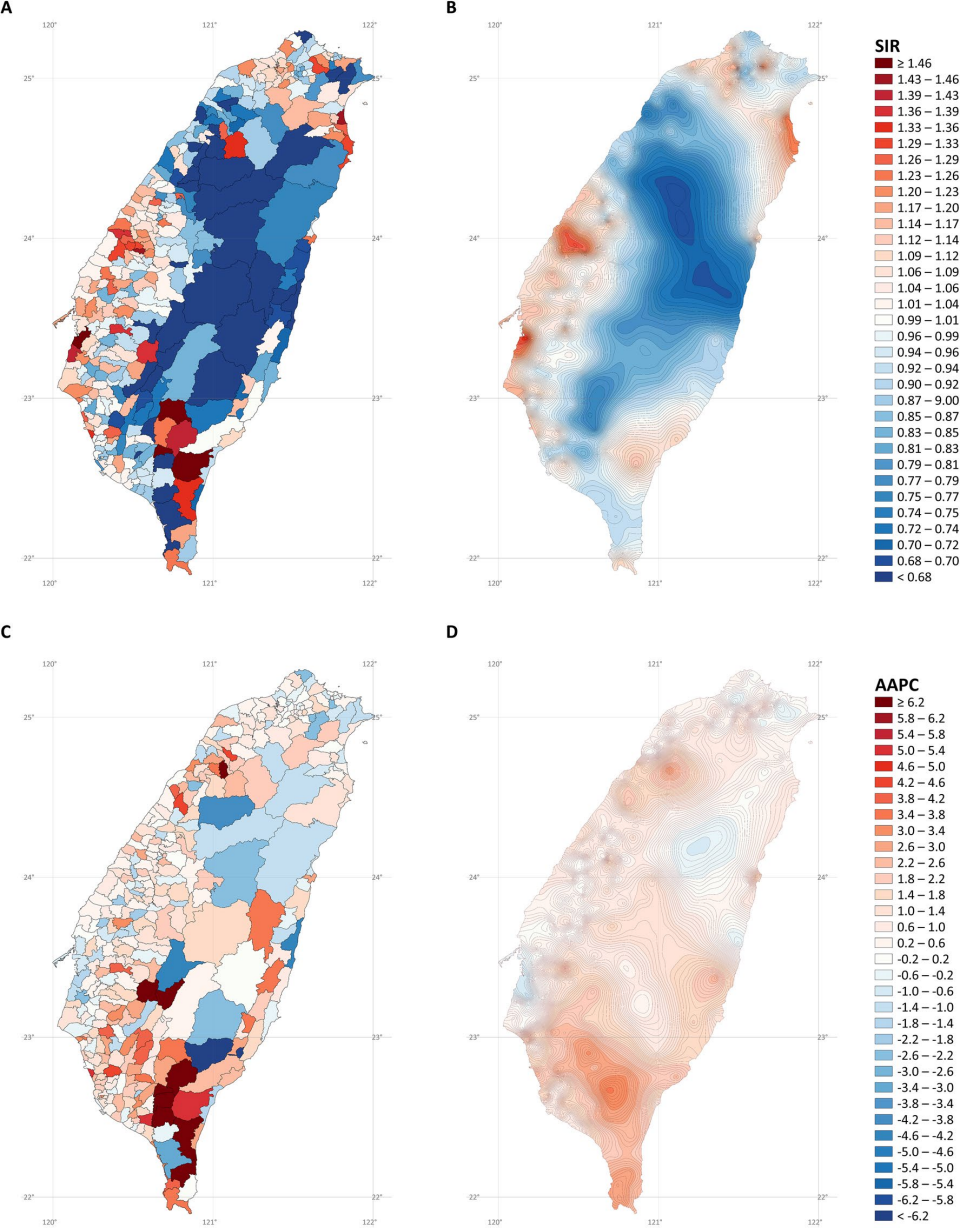
Maps of lung cancer in males (**A)** standardized incidence ratios; (**B)** stabilized kriged standardized incidence ratios; (**C)** average annual percent changes; (**D)** stabilized kriged average annual percent changes). The map was created by R version 3.5.2 with the ggplot2 package (https://cran.r-project.org/web/packages/ggplot2/index.html).

Figure 4 presents the SIR and AAPC maps of lung cancer in men. It is challenging to interpret the overall trend from the noisy SIR (Fig. 4A) and AAPC (Fig. 4C) maps without stabilized kriging. However, after stabilized kriging, we identified several hotspots of incidence rates (Fig. 4B) in the northern, western, and southwestern coastal cities; northeastern coastal towns; southeastern cities; and mountainous areas of Taiwan. We also identified several hotspots exhibiting rapid rate increases in the northwestern and southern coastal towns, southern cities, and mountainous regions (Fig. 4D).

**Figure 5:**
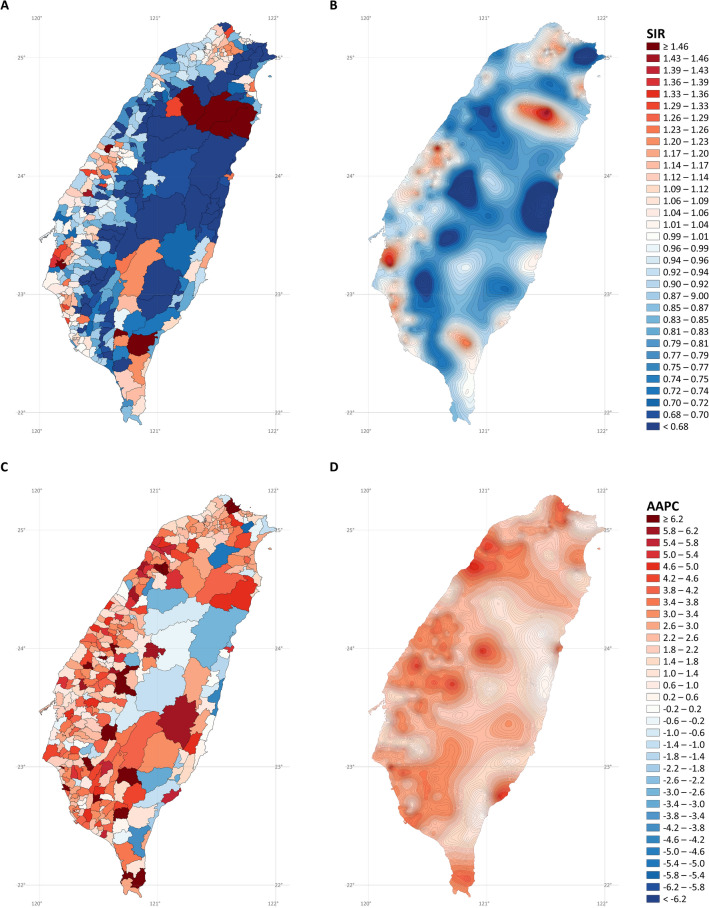
Maps of lung cancer in females (**A)** standardized incidence ratios; (**B)** stabilized kriged standardized incidence ratios; (**C)** average annual percent changes; (**D)** stabilized kriged average annual percent changes). The map was created by R version 3.5.2 with the ggplot2 package (https://cran.r-project.org/web/packages/ggplot2/index.html).

Figure 5 depicts the SIR and AAPC maps of lung cancer in women. Again, it is challenging to interpret the overall trends from the noisy SIR (Fig. 5A) and AAPC (Fig. 5C) maps without stabilized kriging. After stabilized kriging, we identified several hotspots of incidence rates (Fig. 5B) in the northern, western, and southwestern cities, northeastern coastal towns and mountainous areas, and southeastern mountainous regions of Taiwan. We also identified several hotspots in the northern, western, and southern coastal cities and towns and rural towns in central Taiwan, where rates increased rapidly (Fig. 5D).

**Figure 6:**
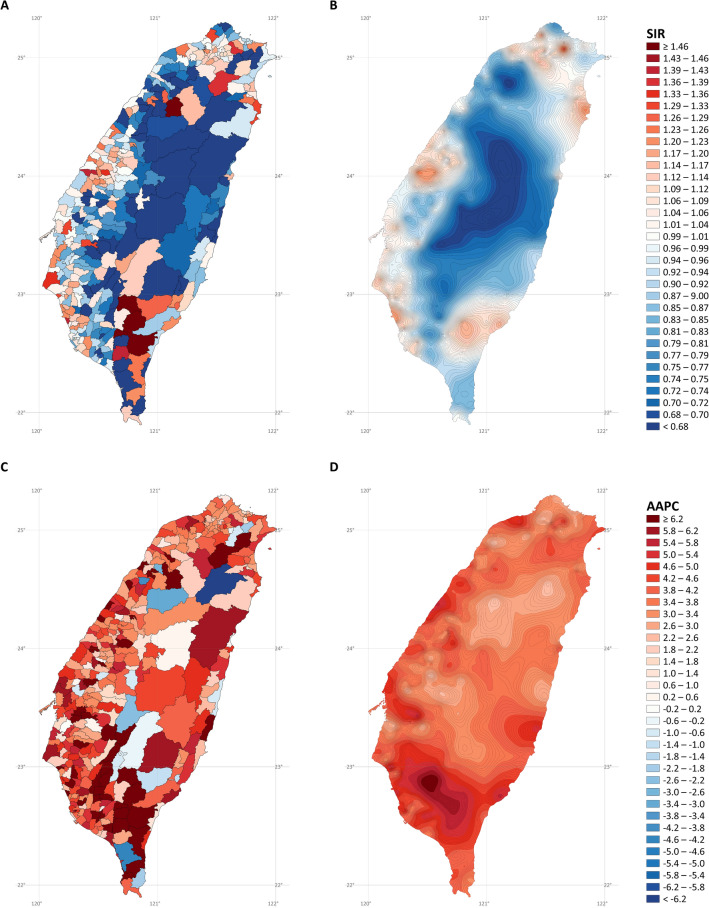
Maps of lung adenocarcinoma cancer in males (**A)** standardized incidence ratios; (**B)** stabilized kriged standardized incidence ratios; (**C)** average annual percent changes; (**D)** stabilized kriged average annual percent changes). The map was created by R version 3.5.2 with the ggplot2 package (https://cran.r-project.org/web/packages/ggplot2/index.html).

Figure 6 presents the SIR and AAPC maps of lung adenocarcinoma in men. The hotspots were similar to those for overall lung cancer in men. Incidence rate hotspots (after stabilized kriging; Fig. 6B) were found in the northern, western, and southwestern coastal cities, northeastern coastal towns, southeastern cities, and mountainous areas of Taiwan. Hotspots of increasing rates (after stabilized kriging; Fig. 6D) were discovered in the western and eastern coastal cities and towns and southern cities and mountainous areas of Taiwan.

**Figure 7:**
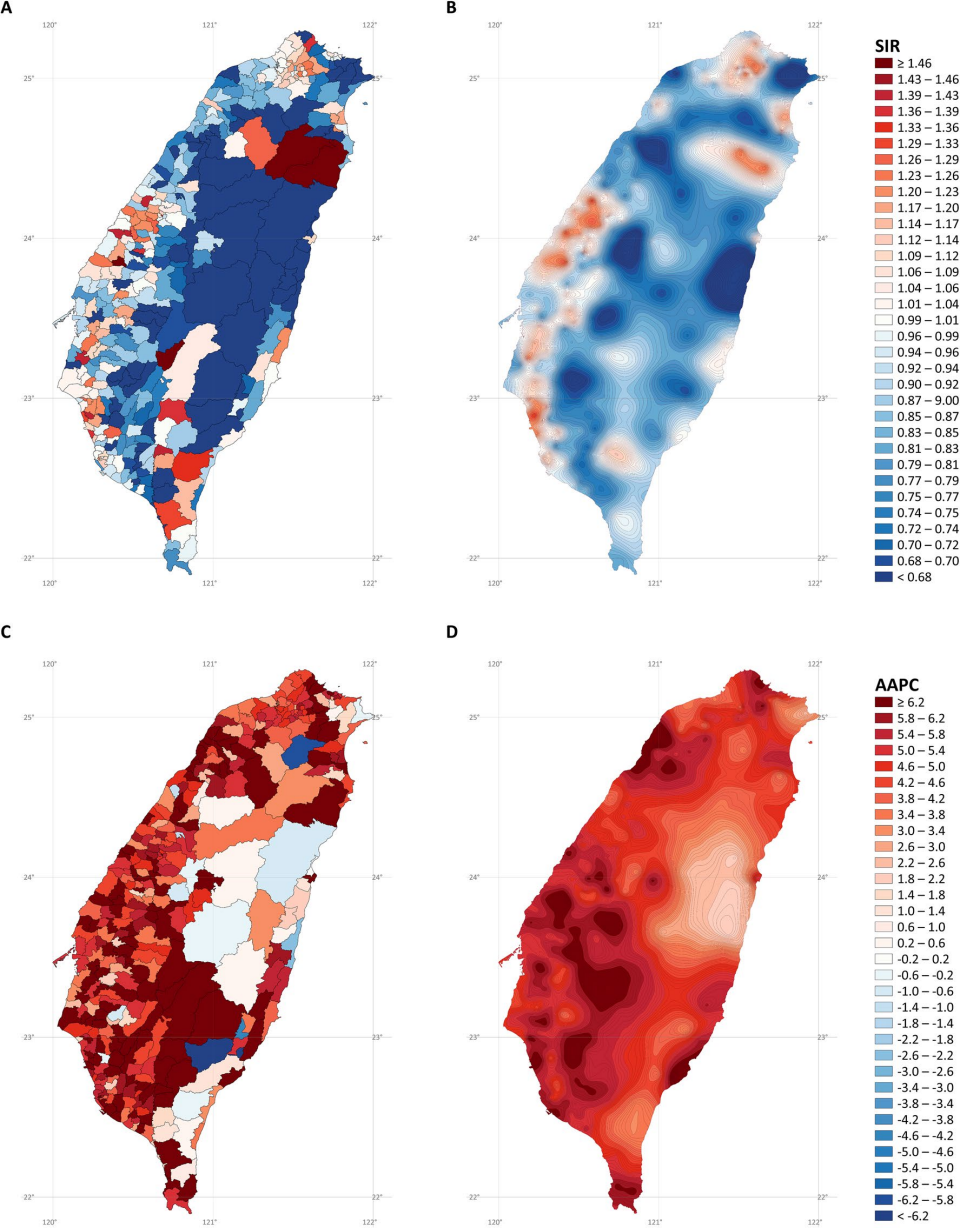
Maps of lung adenocarcinoma cancer in females (**A)** standardized incidence ratios; (**B)** stabilized kriged standardized incidence ratios; (**C)** average annual percent changes; (**D)** stabilized kriged average annual percent changes). The map was created by R version 3.5.2 with the ggplot2 package (https://cran.r-project.org/web/packages/ggplot2/index.html).

Figure 7 shows the SIR and AAPC maps of lung adenocarcinoma in women. The hotspots were similar to those for overall lung cancer in women. Incidence rate hotspots (after stabilized kriging; Fig. 7B) were found in the northern, western, and southwestern coastal cities, northeastern coastal towns, mountainous areas, and southern mountainous areas of Taiwan. Hotspots of increasing rates (after stabilized kriging; Fig. 7D) were observed in northern, western, southern, and southeastern coastal cities and towns; southern mountainous areas; and rural towns in central Taiwan.

**Figure 8:**
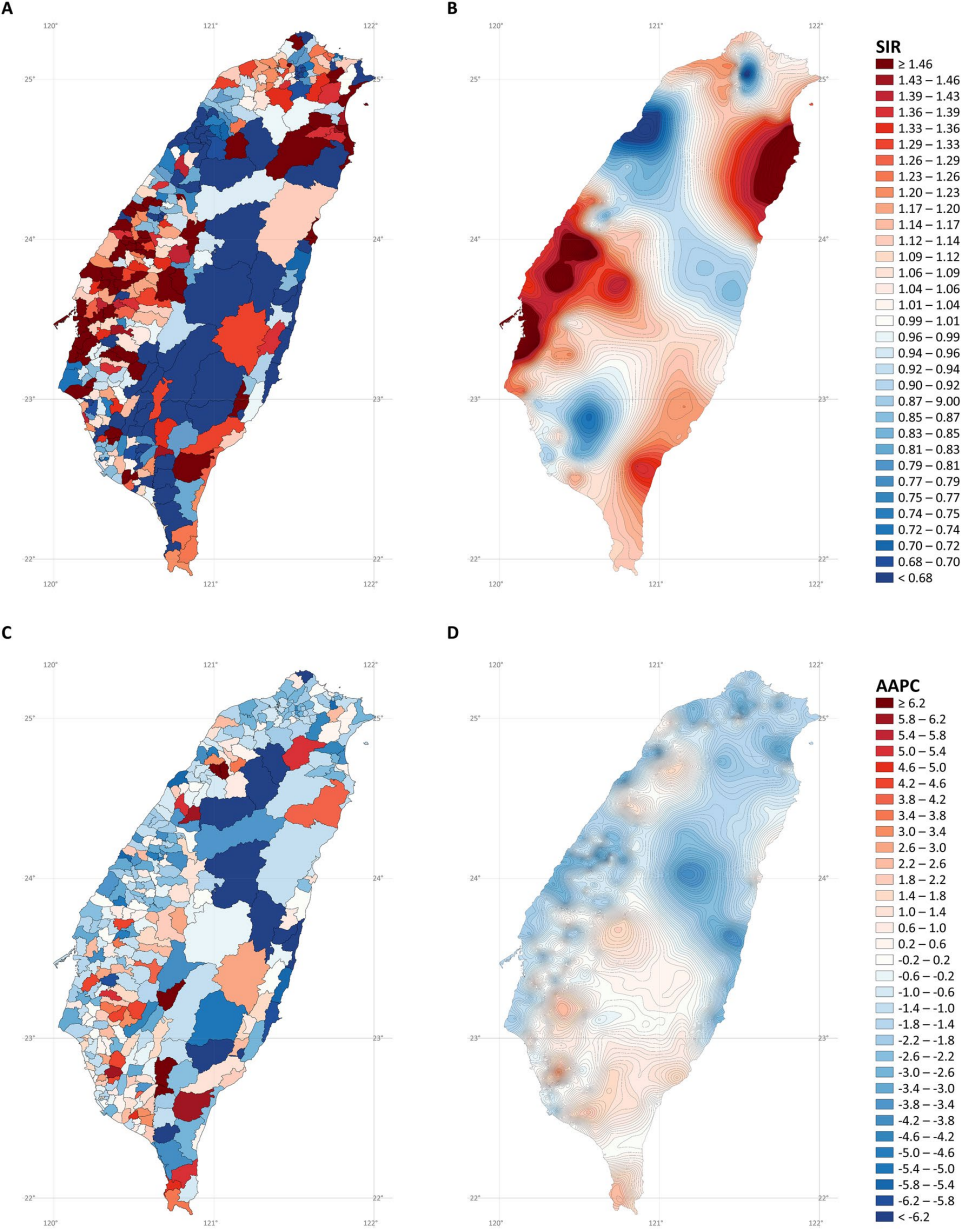
Maps of lung squamous cell carcinoma cancer in males (**A)** standardized incidence ratios; (**B)** stabilized kriged standardized incidence ratios; (**C)** average annual percent changes; (**D)** stabilized kriged average annual percent changes). The map was created by R version 3.5.2 with the ggplot2 package (https://cran.r-project.org/web/packages/ggplot2/index.html).

Figure 8 displays the SIR and AAPC maps of lung squamous cell carcinoma in men. After stabilized kriging, incidence rate hotspots (Fig. 8B) were identified in the southwestern and northeastern coastal cities and towns. Increased rates (after stabilized kriging; Fig. 8D) were found in northwestern, southwestern, southern, and eastern coastal cities and rural towns in central Taiwan.

We could not identify any hotspot for female lung squamous cell carcinoma because the number of cases was insufficient (Supplement 7).

## Discussion

We discovered that the lung squamous cell carcinoma incidence rate in men in Taiwan decreased consistently from 1997 to 2017, particularly among recent birth cohorts. Smoking is a major risk factor for lung squamous cell carcinoma^3,4^. In Taiwan, smokers are predominantly male. After Taiwan implemented the Tobacco Hazards Prevention Act in 1997, smoking prevalence in men decreased considerably^7^. A corresponding decrease was observed in men’s long-term lung squamous cell carcinoma incidence (Fig. 1 and Supplement 8). High lung squamous cell carcinoma incidence rates in men were found mainly in Taiwan’s northwestern and northeastern coastal areas. The prevalence of smoking among men in northeastern regions is also high (Supplement 9). People in southwestern (Budai, Yizhu, Beimen, and Xuejia) and northeastern (Jiaoxi, Wujie, Zhuangwei, and Dongshan) coastal areas (Supplement 10) consumed arsenic-contaminated water from artesian wells before the establishment of the tap water system in the 1960s. Smokers exposed to high arsenic concentrations have an 11-fold higher risk of lung cancer (primarily squamous cell and small cell carcinomas) than nonsmokers exposed to low concentrations of arsenic^17,18^. In the southwestern and northeastern coastal areas, men’s lung squamous cell carcinoma started high but decreased. Supplement 11 shows that the decreasing trends of the birth cohorts were more substantial than those of Taiwan overall; because relevant cases were scarce, only the 1972 and earlier birth cohorts were analyzed. The findings indicate the positive effects of the proliferation of the tap water system in Taiwan.

Smoking prevalence in women in Taiwan was low (< 6%), and it has further decreased in recent years^7^. The lung squamous cell carcinoma incidence rate in women decreased consistently from 1997 to 2017—similar to that in men (Fig. 1 and Supplement 8). The decreasing trend may also be related to a decrease in ETS exposure. ETS inhalation—secondhand or passive smoking—can lead to cancer and thus increase lung cancer incidence rates^19,20^. ETS exposure is primarily familial, and female nonsmokers exposed to ETS have a higher risk of lung squamous cell carcinoma than those without ETS exposure^21^. Nonsmoking women exposed to a smoking environment since childhood have a 70% higher risk of lung cancer than nonsmoking women who were not exposed^22^. In addition, nonsmoking women exposed to their husbands’ ETS have a 2.2-fold higher risk of lung cancer than unexposed^22^. Moreover, ETS exposure increases lung adenocarcinoma risk^23,24^. However, the specific relationships between ETS exposure and histologic types of lung cancer remain unclear^25–27^. This study could not identify hotspots for lung squamous cell carcinoma in women because of the scarcity of cases.

In Taiwan, the incidence rates of lung adenocarcinoma in both sexes increased from 1997 to 2017, whereas those of lung squamous cell carcinoma decreased. Similar trends have been observed in other Asian countries, including South Korea^28^. In Taiwan, the incidence rates of lung adenocarcinoma in both sexes also increased in more recent birth cohorts. The finding contrasts those in the United States, Australia, and Denmark, where the incidence rates of lung adenocarcinoma increased only slowly or started to decline in recent birth cohorts^29,30^. The geographic distributions of lung adenocarcinoma incidence rates in Taiwan were similar for men and women. SIR hotspots were found mainly in the northern, western, and southern coastal cities, northeastern coastal towns, and eastern and southern cities and mountainous areas of Taiwan. Studies have found a dose–response relationship between air pollution and lung adenocarcinoma incidence rates^31–36^. The SIR hotspots of lung adenocarcinoma in Taiwan were correlated with the geographic distribution of air pollutants (including < 2.5- and < 10-μm particulate matter, SO_2_, NO_x_, and O_3_)^32^. The incidence rates of lung adenocarcinoma were lower in southern Taiwan during the early periods, but they eventually increased more rapidly than those in northern Taiwan in more recent years. The finding was probably because the air quality was worse in northern and southern Taiwan in the early period; however, in recent years, air quality has improved in the north and deteriorated in the south^8^. The SIR hotspots of lung adenocarcinoma in Taiwan were also correlated with the geographic distributions of heavy-metal soil contamination with arsenic, copper, nickel, and zinc^37^.

In Asian populations, lung adenocarcinoma is predominant in women and nonsmokers^8,38,39^. Asian women are often exposed to cooking oil fumes that contain potential carcinogens^40^. A study found that in Taiwan, Chinese-food chefs certified for more than five years had a 2.3-fold higher risk of lung adenocarcinoma than nonchefs had—with the majority (70%) of Chinese-food chefs being female^41^. Moreover, women who did not use fume extractors for home cooking were discovered to have a 3.5–12-fold higher lung cancer risk than those who did^42^. In the current study, lung adenocarcinoma incidence rates in Taiwanese women increased rapidly in recent birth cohorts. Moreover, early-stage lung adenocarcinoma has grown over the years in proportion^43^. This finding may be partly attributable to the recent increase in the number of nonsmokers who undergo low-dose computed tomography screening for lung cancer in Taiwan^44,45^.

Lung cancer incidence rates in both sexes in Taiwan increased from 1997 to 2017, particularly among women. The increasing trend was initially slower for both sexes in early birth cohorts before accelerating. The rapidly rising trends can be contrasted with other common cancers in Taiwan, which have also rapidly increasing incidence rates but have more “benign” birth cohort trends. For instance, breast cancer—a leading cancer type in women—increased in early birth cohorts but decreased in recent ones^46^. Moreover, the risk of oral cancer—the fourth most common cancer in men—increased rapidly in early birth cohorts and declined in recent birth cohorts^47^. The SIR hotspots of lung cancer were mainly in the northern, western, and southwestern coastal areas, northeastern coastal towns, and southeastern mountainous areas of Taiwan. For men, the AAPC hotspots of lung cancer were mainly in the northwestern and southern regions, whereas those for women were in the northeastern, eastern, and central areas. Similar findings were observed by Hsu et al.^48^; however, they did not explore the geographic distribution of histological types of lung cancer.

This study obtained incident lung cancer cases from the Taiwan Cancer Registry, a national database with high completeness and accuracy^11,12^. The data on the administrative area of residence was based on household registration. In Taiwan, the agreement between household registration addresses and actual addresses is approximately 80%^49^. The current study was ecological, and thus any inference is prone to ecological fallacy. Further research using individual-level data is therefore warranted. We employed a direct age standardization method (based on the WHO 2000 Standard) to establish secular trends and an indirect age standardization method using SIR (based on Taiwan’s age-specific incidence rates) to analyze geographic variation. Although the direct method enabled comparisons of populations and periods, it produced statistically unstable results (with large random errors). We applied the more stable indirect method (SIR) to study geographic variations because some areas had no lung cancer cases. The harmonically weighted ratio (HWR)^50^, an alternative age standardization index, is statistically stable and allows for comparisons between populations and across time. Maps based on SIR and HWR were almost identical (Supplement 12), suggesting that SIR was appropriate. In addition, we used the stabilized kriging method to account for geographically heterogeneous variance. The SIR in an administrative area with a small population (and thus high variance) was shrunken to one (the null value) considerably, whereas the SIR in an administrative area with a large population was shrunken minimally; consequently, we obtained a stabilized map with smooth contour lines. Finally, we divided the period into two intervals (1997–2007 and 2008–2017; Supplement 13); the AAPC hotspots in the two intervals were nearly identical.

In conclusion, in Taiwan, lung squamous cell carcinoma incidence rates declined for both sexes between 1997 and 2017, but those of lung adenocarcinoma increased. In the future, lung squamous cell carcinoma incidence rates may continue to decrease. The increased incidence rates of lung adenocarcinoma may be related to indoor and outdoor air pollution. In some areas of Taiwan, lung cancer incidence rates were low in the past and increased rapidly over time; these areas, which warrant particular attention, include the northwestern and southern coasts and mountainous regions of Taiwan.

## Supplementary Information

Supplementary Information.

## Data Availability

The Taiwan Cancer Registry database is only available if any research institute has obtained permission from the Department of Statistics, Ministry of Health and Welfare in Taiwan. For the cancer-specific indices of the Taiwan Cancer Registry database, please refer to the link: https://cris.hpa.gov.tw/ (traditional Chinese only). To apply the Taiwan Cancer Registry database usage, please contact the corresponding author (e-mail: wenchung@ntu.edu.tw) for more information.
