# Supplementary material for: Incidence trends and spatial distributions of lung adenocarcinoma and squamous cell carcinoma in Taiwan
Source: Sci Rep. 2023 Jan 30;13:1655. doi: 10.1038/s41598-023-28253-4 (PMC9887070; doi:10.1038/s41598-023-28253-4)

Supplement 1. International Classification of Diseases for Oncology, 3rd edition (ICD-O-3) morphology codes for lung cancer histological types.

	**ICD-O-3 morphology codes**
Adenocarcinoma	8050, 8130, 8140, 8141, 8143, 8144, 8145, 8201, 8211, 8213, 8230, 8250, 8251, 8252, 8253, 8254, 8255, 8256, 8257, 8260, 8262, 8263, 8265, 8290, 8310, 8320, 8323, 8333, 8480, 8481, 8490, 8503, 8550, 8551, 8552, 8572
Squamous cell carcinoma	8052, 8070, 8071, 8072, 8073, 8074, 8075, 8076, 8083, 8084
Large cell carcinoma	8003, 8012, 8013, 8014, 8020, 8021, 8082, 8123
Small cell carcinoma	8002, 8041, 8042, 8043, 8044, 8045
Other carcinoma	(Other specified carcinoma)<br>8023, 8046, 8120, 8122, 8145, 8500, 8507, 8574, 8576, 9100<br>(Unspecified carcinoma)<br>8010

Supplement 2. Calculating the average annual percent change in each administrative area.

We assume the observed numbers of incident lung cancer cases over time in an administrative area follow the Poisson distribution. Let the logarithms of the expected values over time be the offsets and impose a Poisson regression analysis as below:

$$\log(\text{observed value}) = \ln(\text{expected value}) + \beta_0 + \beta_1 \times \text{year}.$$

The average annual percent change (AAPC) in that administrative area is then $\text{AAPC} = 100 \times \hat{\beta}_1$. Noteworthy, here we use the "sympercent" by Cole (Cole TJ. Sympercents: symmetric percentage differences on the 100 log(e) scale simplify the presentation of log-transformed data. Stat Med 2000;19:3109-3125). When $\hat{\beta}_1 \approx 0$, the difference between the sympercent and the traditional percentage difference is slight:

$$\text{AAPC} = 100 \times \hat{\beta}_1 \approx 100 \times \left(e^{\hat{\beta}_1} - 1\right) = 100 \times \frac{e^{\hat{\beta}_0 + \hat{\beta}_1 \times (\text{year}+1)} - e^{\hat{\beta}_0 + \hat{\beta}_1 \times \text{year}}}{e^{\hat{\beta}_0 + \hat{\beta}_1 \times \text{year}}}.$$

But otherwise, the traditional percentage difference is constrained to have a lower limit (the lower limit is -100), which is unreasonable. In contrast, the sympercent is symmetric and is unconstrained in either direction.

Supplement 3. The proportion of lung cancer histologic types from 1997 to 2016 (A: males; B: females).

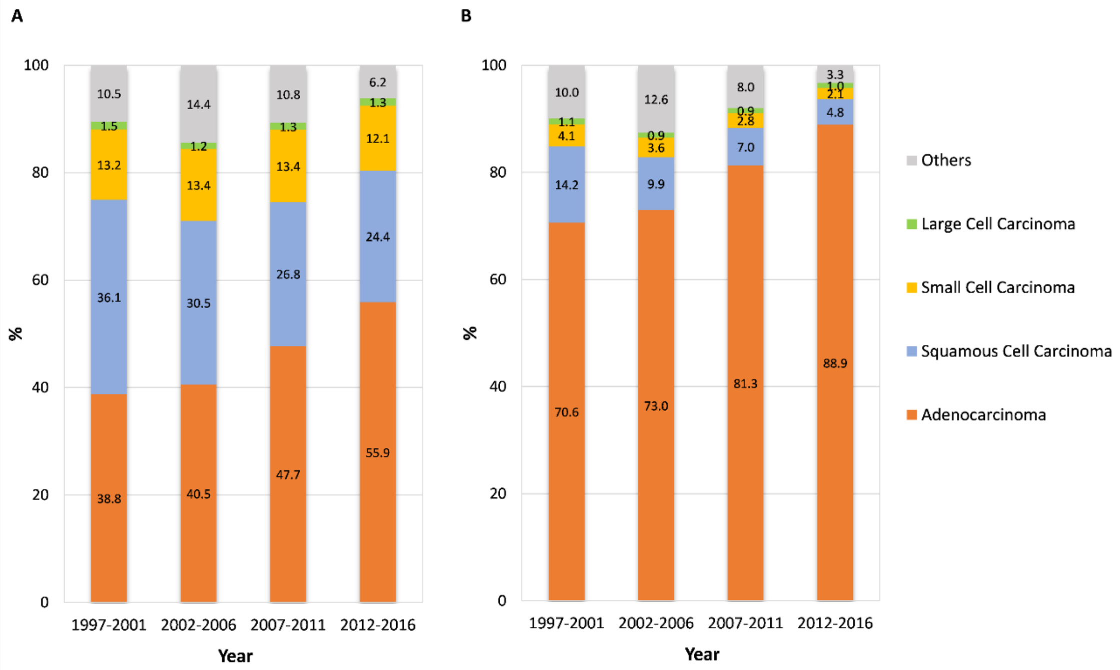

Supplement 4. Incidence rates of lung adenocarcinoma by age, period, and cohort (A: males; B: females).

A

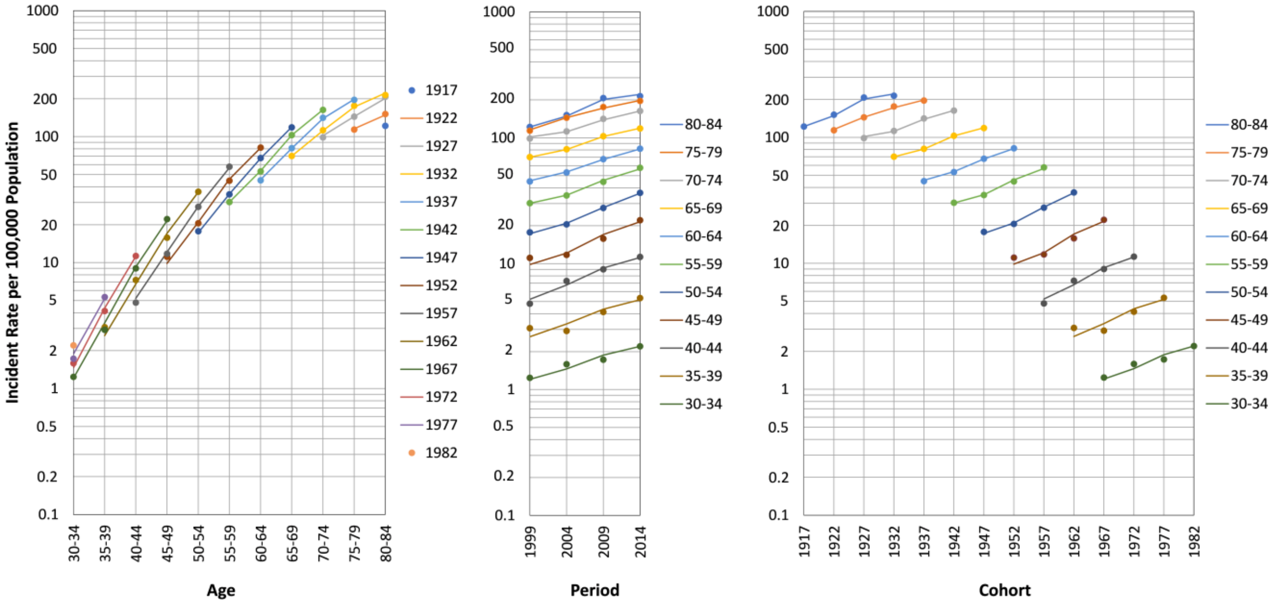

B

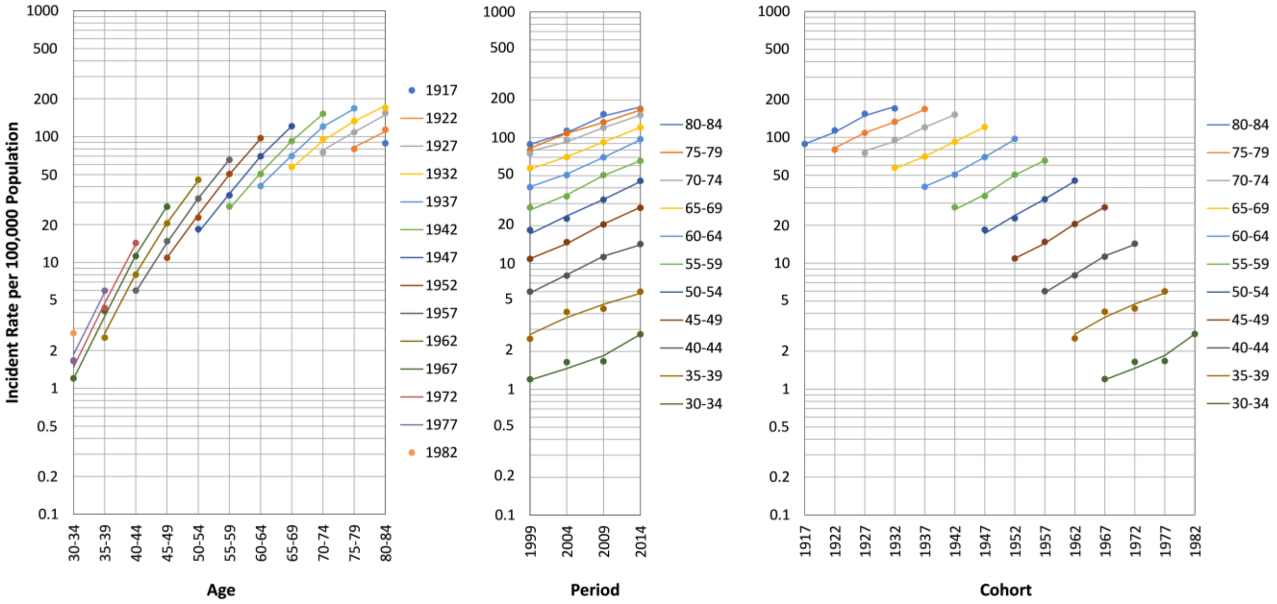

Supplement 5. Incidence rates of lung squamous carcinoma by age, period, and cohort (A: males; B: females).

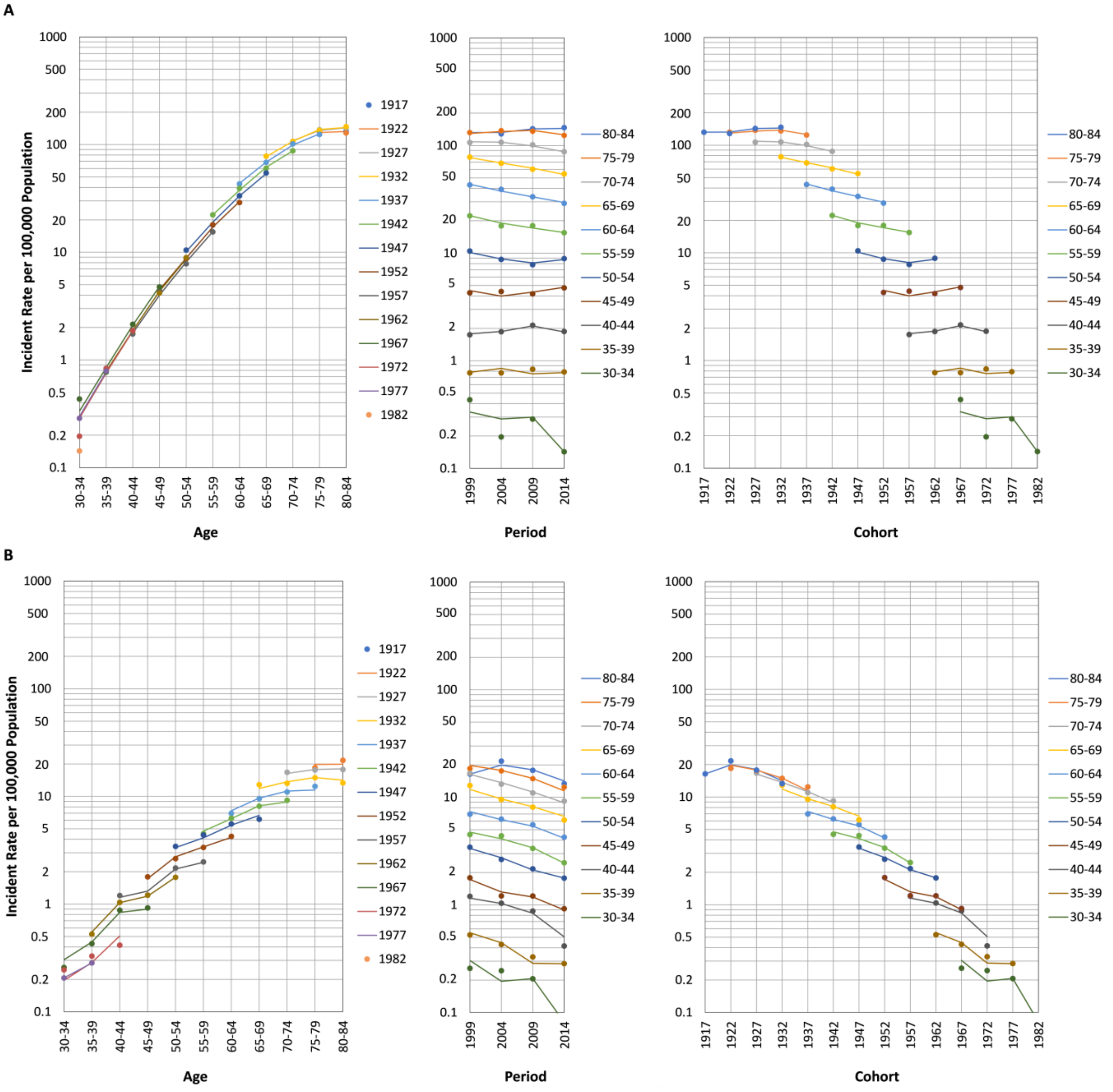

Supplement 6. The average annual percent changes of lung cancer incidence by sex and age groups (30-34, 35-39, …, 80-84) from 1997 to 2017.

Age groups	Histological types					
	All types		Adenocarcinoma		Squamous cell carcinoma	
	AAPC	95% CI	AAPC	95% CI	AAPC	95% CI
**Male**						
30-34	**1.5**	**(0.6 to 2.4)**	**3.5**	**(2.6 to 4.4)**	**-4.1**	**(-7.7 to -0.4)**
35-39	**2.3**	**(1.2 to 3.4)**	**4.1**	**(2.8 to 5.4)**	-0.6	(-3.0 to 1.8)
40-44	**3.4**	**(2.6 to 4.2)**	**5.3**	**(4.4 to 6.3)**	0.8	(-1.3 to 2.8)
45-49	**3.1**	**(2.5 to 3.7)**	**5.3**	**(4.5 to 6.1)**	0.4	(-0.9 to 1.7)
50-54	**2.5**	**(2.1 to 2.9)**	**5.3**	**(4.7 to 5.8)**	-0.8	(-2.6 to 1.0)
55-59	**1.2**	**(0.9 to 1.4)**	**4.5**	**(3.9 to 5.0)**	**-1.9**	**(-2.6 to -1.3)**
60-64	**0.6**	**(0.3 to 0.9)**	**4.2**	**(3.8 to 4.5)**	**-2.5**	**(-3.1 to -1.9)**
65-69	-0.1	(-0.4 to 0.2)	**3.5**	**(3.1 to 3.9)**	**-2.4**	**(-3.1 to -1.7)**
70-74	0.3	(-0.1 to 0.7)	**3.4**	**(2.8 to 3.9)**	**-1.2**	**(-2.1 to -0.3)**
75-79	0.5	(-0.1 to 1.1)	**5.0**	**(2.9 to 7.2)**	-0.9	(-2.3 to 0.5)
80-84	**0.9**	**(0.3 to 1.6)**	**3.6**	**(2.6 to 4.7)**	0.7	(-0.1 to 1.4)
**Female**						
30-34	**3.7**	**(2.4 to 5.0)**	**5.6**	**(3.7 to 7.5)**	-1.8	(-5.9 to 2.5)
35-39	**3.4**	**(1.9 to 5.0)**	**5.4**	**(3.8 to 7.1)**	**-4.7**	**(-8.3 to -0.9)**
40-44	**3.6**	**(3.0 to 4.2)**	**6.0**	**(5.4 to 6.6)**	**-5.2**	**(-7.9 to -2.5)**
45-49	**4.4**	**(3.9 to 4.9)**	**6.4**	**(5.9 to 6.9)**	**-4.1**	**(-5.8 to -2.3)**
50-54	**4.2**	**(3.0 to 5.4)**	**6.8**	**(6.2 to 7.4)**	**-3.9**	**(-5.5 to -2.2)**
55-59	**4.3**	**(3.8 to 4.8)**	**6.2**	**(5.7 to 6.8)**	**-3.9**	**(-5.5 to -2.2)**
60-64	**3.9**	**(3.6 to 4.3)**	**6.1**	**(5.5 to 6.6)**	**-3.0**	**(-4.2 to -1.8)**
65-69	**3.1**	**(2.1 to 4.1)**	**5.3**	**(4.9 to 5.7)**	**-4.5**	**(-5.8 to -3.3)**
70-74	**2.0**	**(1.5 to 2.4)**	**4.5**	**(4.0 to 5.1)**	**-3.7**	**(-4.6 to -2.9)**
75-79	**1.8**	**(1.3 to 2.2)**	**4.9**	**(4.3 to 5.6)**	**-3.0**	**(-4.1 to -1.8)**
80-84	**1.2**	**(0.3 to 2.1)**	**4.2**	**(3.5 to 5.0)**	-1.7	(-4.5 to 1.2)

Abbreviations: AAPC: average annual percent change; CI: confidence interval.
Bold format numbers: statistical significance at a significance level of 0.05.

Supplement 7. Maps of lung squamous cell carcinoma in females in Taiwan (A: standardized incidence ratios; B: stabilized kriged standardized incidence ratios; C: average annual percent changes; D: stabilized kriged average annual percent changes).

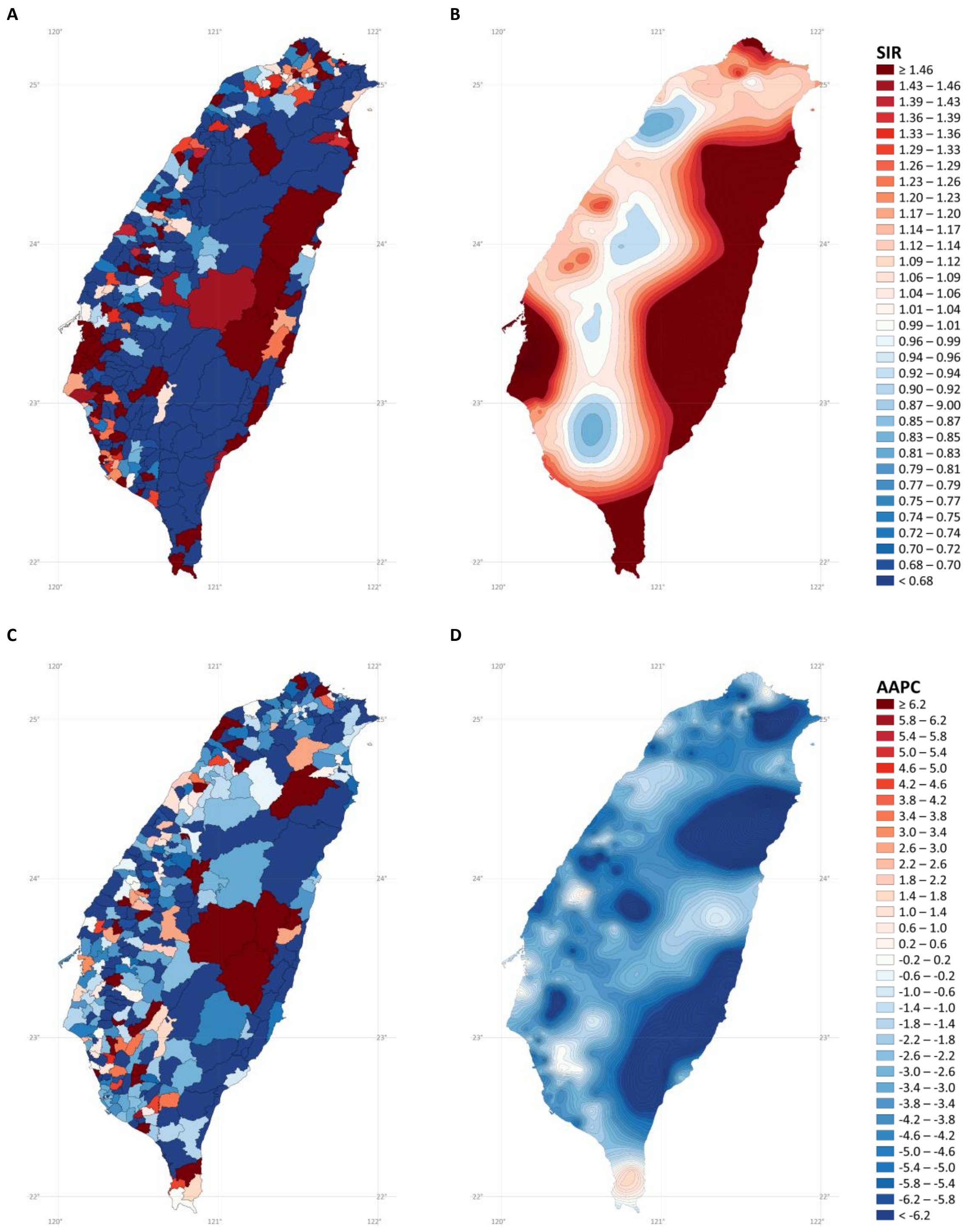

Supplement 8. Association between lung squamous cell carcinoma incidence and smoking prevalence by sex from 1997 to 2017 (LEFT: male; RIGHT: female).

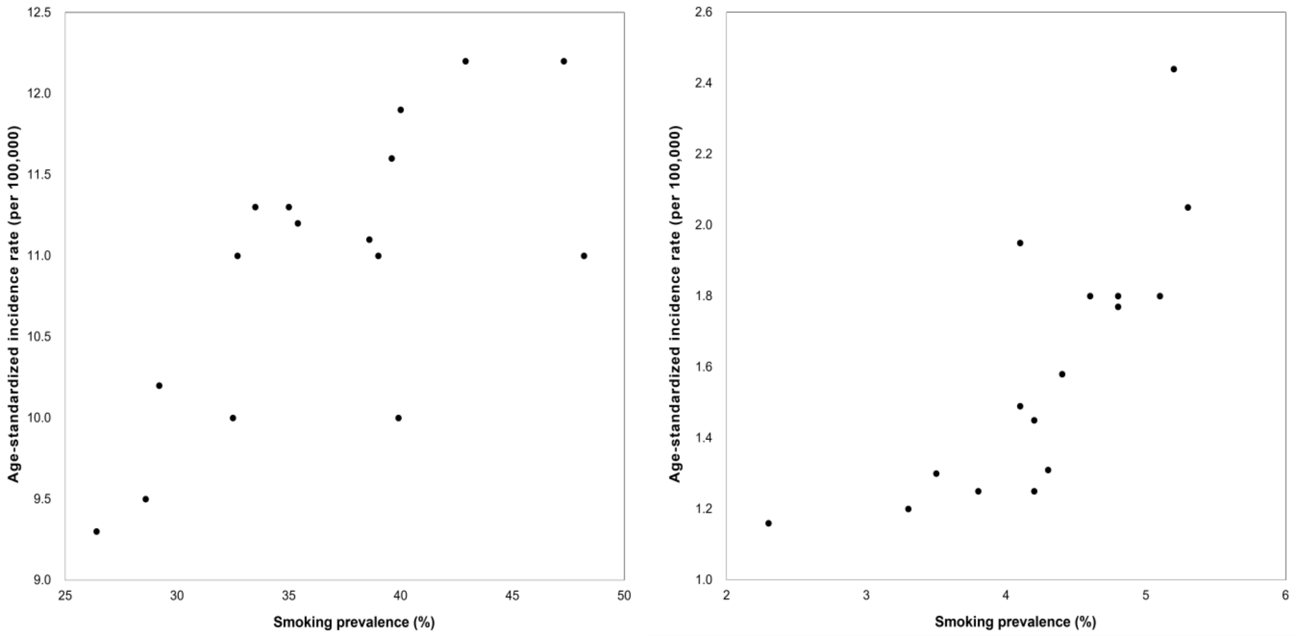

Supplement 9. Smoking prevalence rates in males aged over 18 in Taiwan in 2004.

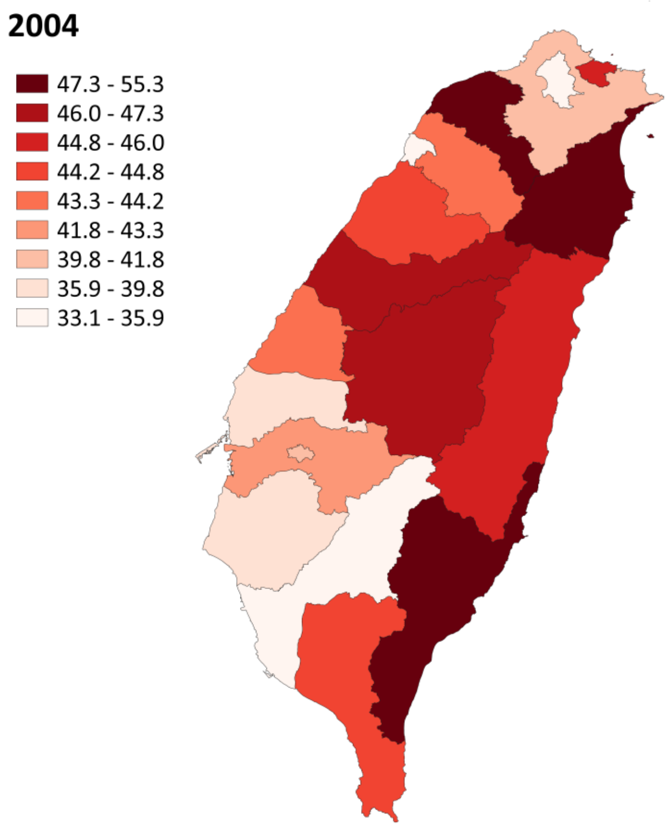

Supplement 10. Areas with arsenic-contaminated water in Taiwan.

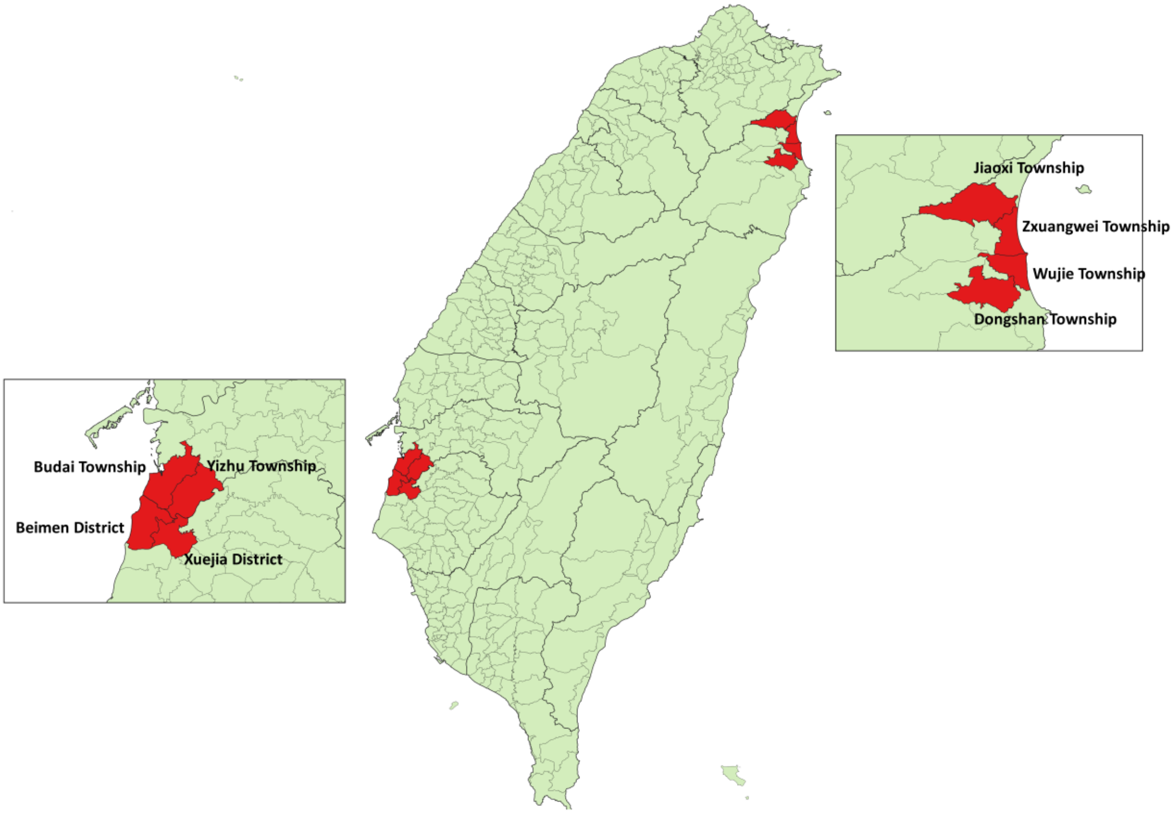

Supplement 11. Cohort effects of lung squamous cell carcinoma in males in different areas.

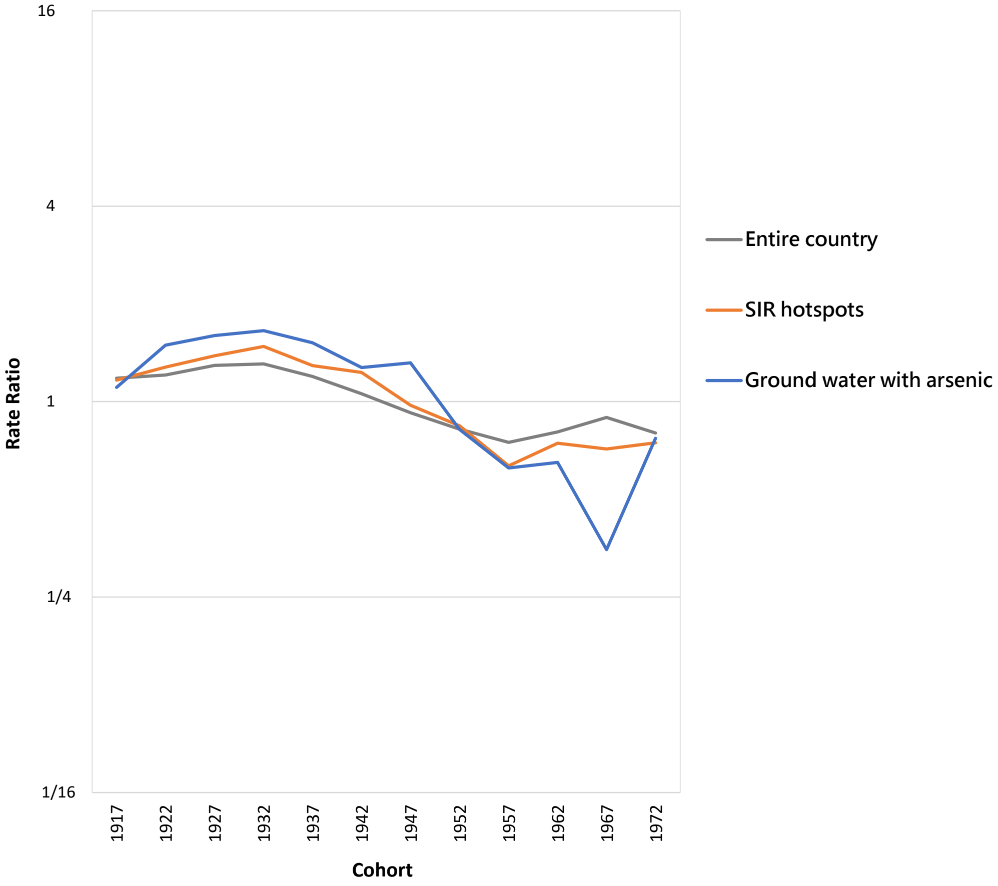

* Areas with arsenic contaminated water (west-sourthern areas: Budai, Yizhu, Beimen, and Xuejia; east-northern areas: Jiaoxi, Wujie, Zhuangwei, and Dongshan).

Supplement 12. Stabilized kriged maps of lung cancer in males and females in Taiwan (A: standardized incidence ratios in males; B: harmonically weighted ratios in males; C: standardized incidence ratios in females; D: harmonically weighted ratios in females).

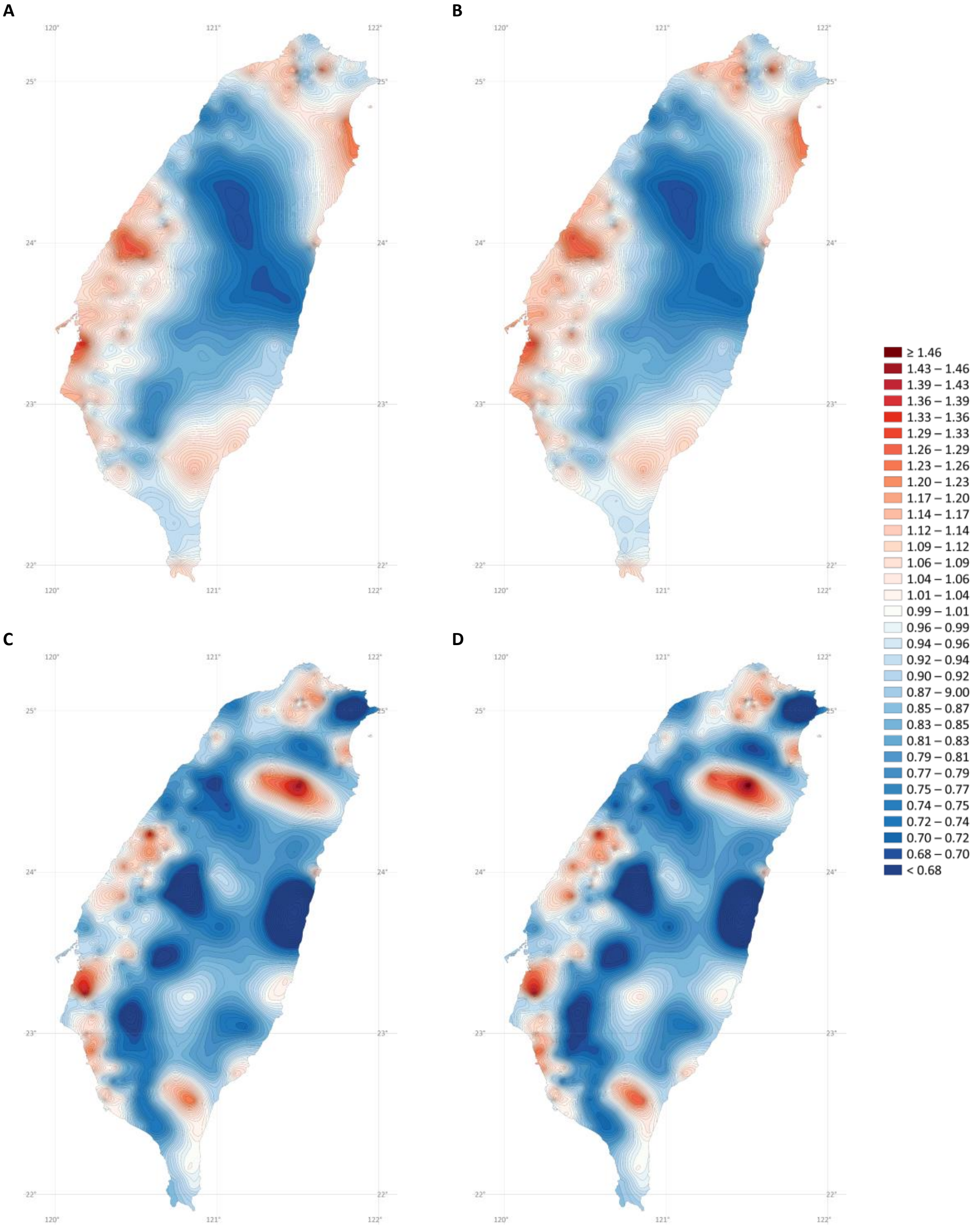

Supplement 13. Stabilized kriged maps of average annual percent changes from 1997 to 2007 and 2008 to 22017 (A: lung cancer in males; B: lung cancer in females; C: lung adenocarcinoma in males; D: lung adenocarcinoma in females; E: lung squamous cell carcinoma in males).

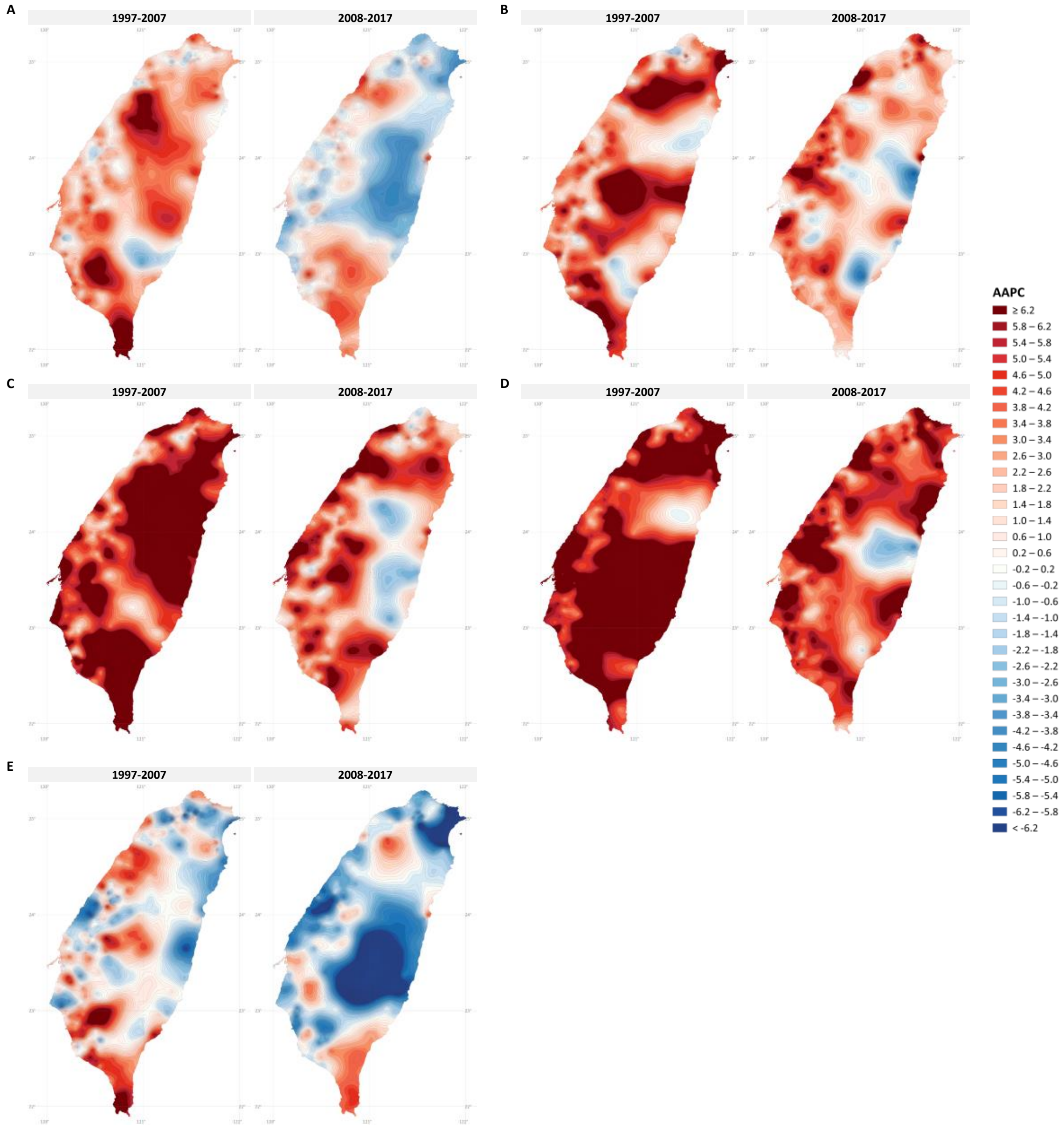

Supplement: Supplementary file 1 — Supplementary Information. [file 41598_2023_28253_MOESM1_ESM.pdf]
